# Rapid *in silico* Design of Potential Cyclic Peptide Binders Targeting Protein-Protein Interfaces

**DOI:** 10.3389/fchem.2020.573259

**Published:** 2020-10-08

**Authors:** Brianda L. Santini, Martin Zacharias

**Affiliations:** Physics Department T38, Technical University of Munich, Garching, Germany

**Keywords:** protein-protein complexes, protein interaction inhibition, protein binding modulation, cyclo peptide design, drug design with cyclo-peptides, rational cyclo peptide binders

## Abstract

Rational design of specific inhibitors of protein-protein interactions is desirable for drug design to control cellular signal transduction but also for studying protein-protein interaction networks. We have developed a rapid computational approach to rationally design cyclic peptides that potentially bind at desired regions of the interface of protein-protein complexes. The methodology is based on comparing the protein backbone structure of short peptide segments (epitopes) at the protein-protein interface with a collection of cyclic peptide backbone structures. A cyclic peptide that matches the backbone structure of the segment is used as a template for a binder by adapting the amino acid side chains to the side chains found in the target complex. For a small library of cyclic peptides with known high resolution structures we found for the majority (~82%) of 154 protein-protein complexes at least one very well fitting match for a cyclic peptide template to a protein-protein interface segment. The majority of the constructed protein-cyclic peptide complexes was very stable during Molecular Dynamics simulations and showed an interaction energy score that was typically more favorable compared to interaction scores of typical peptide-protein complexes. Our cPEPmatch approach could be a promising approach for rapid suggestion of cyclic peptide binders that could be tested experimentally and further improved by chemical modification.

## Introduction

Protein-protein interactions (PPIs) serve as the basis for nearly all biological processes, they play key roles in intercellular communication, cell-to-cell signaling, metabolic and developmental control, and programmed cell death (Fontaine et al., 2015). Abnormal regulation of PPIs results in many human diseases such as cancers, immune disorders, and neurodegenerative diseases. Modulating these aberrant interactions is of clinical relevance, however, targeting PPIs may be challenging because of the intrinsic properties of protein–protein interfaces (Ryan and Matthews, 2005; Villoutreix et al., 2014).

PPI interfaces typically involve large, flat binding sites which are devoid of any major binding pocket (Conte et al., 1999; Bahadur and Zacharias, 2008), making them challenging targets for conventional drug modalities because small molecules generally do not bind to large binding sites with high affinity. Larger molecules with more extended binding surfaces, e.g., monoclonal antibodies, cannot easily cross the cell membrane to reach the targets (Qian et al., 2017). Progress has, however, been made in targeting protein-protein interactions (PPI) by realizing that not all the surface area of a PPI contributes equally to the strength of the interaction between the protein partners (Keskin et al., 2005; Wells and McClendon, 2007; Arkin et al., 2014). Their binding is mediated by strong packing or electrostatic interactions where only a few neighboring amino acids are crucial for binding and recognition. These small areas of disproportionately high-affinity binding at the protein–protein interface are referred to as “hot spots” (Keskin et al., 2005; Metz et al., 2012). Nevertheless, targeting specifically protein–protein interaction hot spots still tends to require complex drug molecules (Fry, 2006).

A promising approach for PPI targeting is the design of molecules that closely mimic epitopes, i.e., binding segments, of a partner protein found as part of the interface. These molecules are typically derived from existing peptides and tend to conserve a protein-like chain, but with its chemical structure modified to adjust the molecular properties to become more drug-like. Following this strategy, many investigators have turned their attention to macrocycles, particularly cyclic peptides, as potential PPI inhibitors (Driggers et al., 2008; Marsault and Peterson, 2011; Mallinson and Collins, 2012). Cyclic peptides are among the most promising PPI modulators owing to their ability to potentially bind to large surfaces with reasonable affinity and specificity and due to the enhanced stability and bioavailability as compared to linear peptides (Grauer and König, 2009; Gavenonis et al., 2014; Nevola and Giralt, 2015). The advantages of cyclic peptides in terms of conformational rigidity and proteolytic resistance can also be further enhanced by modifying the peptide backbone. Proteases act upon cleavage sites in the peptide sequence, which may be at the N or C-terminus, or sometimes at a particular motif within the peptide. Peptide cyclization often reduces the accessibility to cleavage sites in proteases. There is also evidence that cyclic peptides may have improved membrane permeability, compared with linear peptides, in cases where the cyclic peptide can internally satisfy its hydrogen bonds (Rezai et al., 2006).

Early and recent work has demonstrated the possibility to use cyclic peptides that could bind at protein-protein interface regions and interfere with protein-protein binding (Schreiber and Crabtree, 1992; Dechantsreiter et al., 1999; Sulyok et al., 2001; Shi et al., 2004; Zhang et al., 2014; Siegert et al., 2016; Shin et al., 2017). Identification of potential appropriate cyclic peptides can be achieved using techniques used in small-molecule drug design efforts such as virtual screening and pharmacophore matching approaches (Zhang et al., 2014; Duffy et al., 2015; Qian et al., 2017). In this study, we propose a straight forward automated process for the rapid search and optimization of cyclic peptides as protein-protein interaction inhibitors based on known structures of cyclic peptides. Our main goal is to start with a library of existing cyclic peptides and use them as templates for the construction of molecules that closely mimic the epitopes of a partner protein in PPI complexes. We start our process by characterizing backbones motifs of the backbone of epitopes found on the PPI interfaces and comparing them to backbone motifs in a data base of cyclic peptides. If a backbone match is found, the cyclic peptide structure is superimposed and the corresponding interface amino acids of the cyclic peptides are replaced by those of the binding epitope, to closely mimic the specific interface. The generated cyclo-peptide-protein complexes are refined and evaluated by Molecular Dynamics (MD) simulations and energetic evaluation. In this proof-of-principle study our automated approach is tested on 154 protein-protein complexes. For the majority of ~71% we identify cyclic peptide motifs that result in stable complexes during MD refinement and closely mimic the natural interface epitope.

## Materials and Methods

### Cyclic Peptide Matching (cPEPmatch) Approach

**Figure 1:**
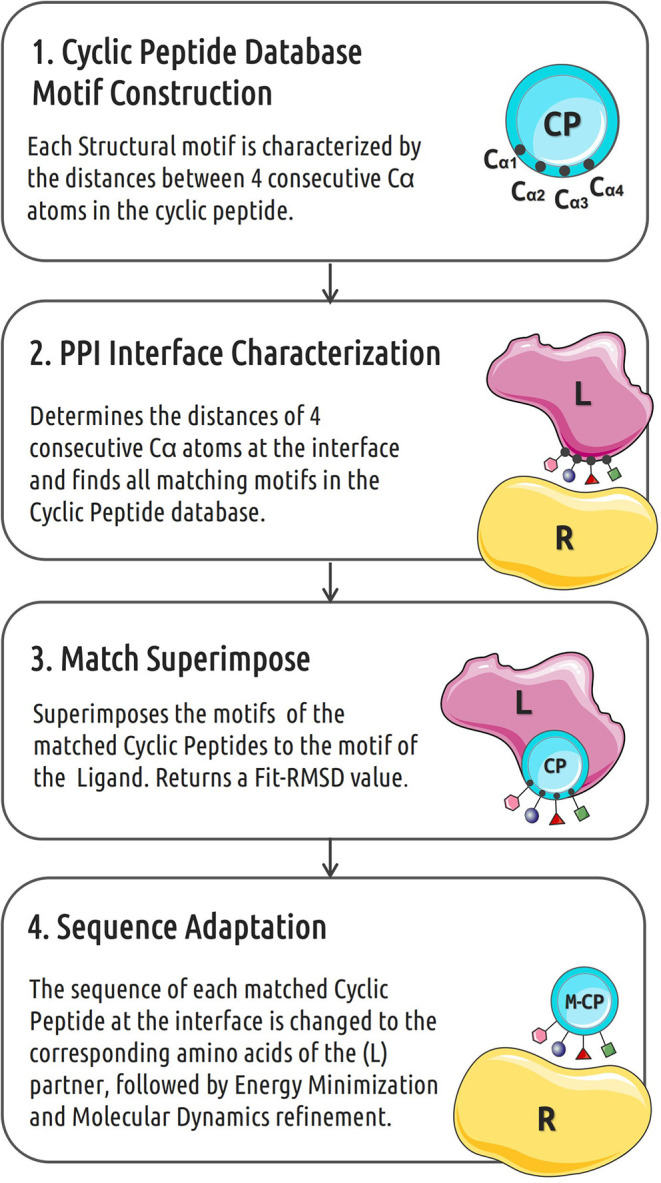
Workflow of the *in silico* cyclic peptide binder construction. CP indicates cyclic peptide, L and R represent the two partners of a protein-protein interaction pair.

Many protein–protein interactions are at least in part mediated by short linear motifs that can be part of α-helices, β-strands or loops (London et al., 2010). The workflow of the cPEPmatch that we propose in this work is based on the idea that the shape of the backbone structure of the PPI interfaces plays a key role in the binding interaction between these short motifs (Kallen et al., 1998). Our cyclic peptide matching approach is divided into 4 main steps (outlined in Figure 1) including a final refinement and evaluation step using the AmberTools18 package (Case et al., 2005). The tool *backbo* characterizes the backbones of each cyclic peptide in terms of distances between backbone atoms of 4 consecutive residues, creating the motifs that are then stored in a data base. A second tool, *int_analysis*, identifies all the neighboring protein residues from the PPI complexes as the interface and, subsequently, characterizes its backbone atom motifs also in sets of four consecutive residues and identifies matching backbone motifs in the cyclic peptide data file. For each protein-protein interface motifs within up to 7 Å distance from the partner protein were considered. For the matching of backbone atom distances of a motif at the protein-protein interface and a corresponding motif in a cyclic peptide an average distance deviation of up to 0.5 Å was used as threshold to accept only sufficiently precise matches (in the following termed F-RMSD). In a third step a cyclic peptide is superimposed on the matching backbone segment of a partner protein to form an appropriate binding placement. This step returns a coordinate file of the cyclic peptide in complex with the receptor partner protein. The side chains of the four consecutive (matching) residues at the interface between cyclic peptide and protein are then replaced by the side chains found in the original protein-receptor complex. Exceptions for residue substitutions are made when the matched residue of the cyclic peptide is one involved in a disulfide bond that is part of the peptide cyclization (not changed). The resulting complex was further refined and evaluated by energy minimization and Molecular Dynamics (MD) simulation (see below).

### Cyclic Peptide Library and Protein-Protein Complexes

Different chemical strategies can be used to create cyclized peptides, including head-to-tail, disulfide, other side-chain to side-chain, and side-chain to terminus bonding (Martins and Carvalho, 2007; Wells and McClendon, 2007; Huigens et al., 2013; Duffy et al., 2015; Kuenemann et al., 2015; Milhas et al., 2016). Incorporating multiple cyclizations to generate peptides that are bicyclic, tricyclic, etc., can provide additional restraints to rigidify the peptide and provide further complexity of design space (Villoutreix et al., 2014; Che, 2019; Duffy et al., 2019). We extracted structures of 30 cyclic peptides that vary in cyclization type and sizes as representative set of cyclic peptides with known well resolved 3D structure. The cyclic peptides structure types have been chosen to represent common portions of protein-protein interaction hot-spots such as α-helices, β-strands, and turns. All structures used were downloaded from the Protein Data Bank (PDB) and a complete list is found in Supplementary Table 1. We applied the method to the analysis of protein-protein complexes chosen from the protein-protein interface (PPI) Benchmark constructed by Tikk and collaborators (Tikk et al., 2010), the complete list of complexes is given in Supplementary Table 2.

### Molecular Dynamics Simulation and MM/GBSA Evaluation

All cyclic peptide-protein complexes were processed for energy minimization and MD simulations using the *tleap* module of Amber18. Protein parameters were retrieved from the ff14SB force field (Maier et al., 2015). The complexes were neutralized by the addition of Na+ or Cl- ions and solvated in an orthorhombic box with a minimum distance to box-boundaries of 10 Å using explicit TIP3P water molecules (Jorgensen et al., 1983). All simulation systems were first energy minimized with the steepest descent method in 2000 steps by using the Amber18 *Sander* module. All subsequent MD simulations were performed with the *pmemd.cuda* module. Initially, the systems were heated up to 310 K in three stages (in 100 K steps). Each stage was simulated for 100 ps and included positional restraints on all non-hydrogen atoms with respect to the starting conformation. Subsequently, positional restraints were gradually reduced from 25 to 0.5 kcal·mol^−1^·A^−2^ in five consecutive simulations of 100 ps at 310 K and at constant pressure of 1 bar. The equilibrated structures served as input for the production runs for each system, with no restraints. Data gathering simulations were carried out for 5 ns. Coordinates were written out every 500 steps. A time step of 2 fs was used and all bonds involving hydrogens were constrained to the optimal length using Shake (Ryckaert et al., 1977). In order to estimate the stability of a bound cyclic peptide the L-RMSD (root-mean-square deviation of all non-hydrogen atoms of the cyclic peptide after best superposition of the receptor binding site on the start structure) was calculated to assess the deviation of peptide ligand along the 5 ns of trajectory for each match. For the superposition on the receptor structure the receptor binding site that is all atoms within 6 Å of any peptide ligand atom was considered.

MM/GBSA (molecular mechanics Generalized Born surface area) binding free energy calculations were carried out to estimate the strength of cyclopeptide-receptor interactions (Chen et al., 2016; Wang et al., 2018). Binding free energies were estimated using 250 snapshots retrieved from the last 2.5 ns of MD simulation production using the MMPBSA.py module in Amber18. GB calculations were carried out using the modified GB model (igb=5) with mbondi2, and α, β, and γ values of 1.0, 0.8, and 4.85, respectively. Dielectric constants for the solvent and the solute were set to 80 and 5, respectively.

## Results and Discussions

Identification of cyclic peptides that bind to the interface region of protein-protein complexes may provide a promising route for modulating PPIs (Qian et al., 2017). In this work, we present an automated rapid workflow (cPEPmatch) that identifies and optimizes cyclic peptides as starting templates to construct modulators of protein-protein interactions. It is, based on the matching of small backbone motifs at protein-protein interfaces and corresponding motifs in cyclic peptides (Figure 1). It has been observed that many protein–protein interaction surfaces are dominated by short segments of peptides (London et al., 2010) and that the constrained backbone often plays a key role in the binding interactions (Kallen et al., 1998).

**Figure 2:**
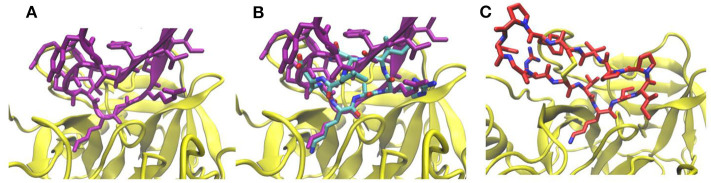
**(A)** Interface structure of the complex (pdb4dg4) of trypsin inhibitor protein (pink) and trypsin (yellow). **(B)** Same view as in **(A)** but with a superimposed cyclic peptide (pdb3avb, atom color coded) and adapted interface side chains after 5 ns MD simulation. **(C)** Complex of sunflower cyclic peptide (red/blue stick model) and trypsin (pdb1sfi).

We first tested the approach on a complex formed by bovine trypsin inhibitor (BPTI) protein and the trypsin protease (pdb4dg4). Our matching approach identified a cyclic peptide backbone that closely resembled the protein backbone of the BPTI protein at the interface (Figures 2A,B) with a root-mean-square deviation (RMSD) of the 4 residue backbone segment of only 0.4 Å with respect to the corresponding segment in the BPTI protein. The side chain replacement resulted in excellent sterical fit closely resembling the original interface structure even after the MD simulation refinement in explicit solvent. The predicted structure of the cyclic-peptide trypsin complex is also in excellent agreement with an experimentally known structure of the sunflower cyclic peptide in complex with trypsin (pdb1sfi, Figure 2C, RMSD of the interface segment <0.5 Å, Table 1). Note, that the sunflower peptide is not included in our cyclic peptide data base. The evaluation of the cyclic peptide interaction with trypsin resulted in a MMGBSA interaction energy of –43.8 kcal·mol^−1^ (Table 1). This is less than what is obtained for the sunflower peptide (–68.8 kcal·mol^−1^) but the sunflower peptide is also significantly larger and forming a more extended interface compared to the generated potential small cyclic peptide binder. The calculated MMGBSA interaction energy for this test case was significantly more favorable than the MMGBSA interaction energy obtained for a typical peptide-protein complex with binding affinities in the micromolar regime (Table 1).

**Table 1:** Performance of the cyclo-peptide matching approach.

**MM/GBSA Comparison**	
Trypsin—Sunflower Cyclic Peptide Inhibitor (1sfi)	–68.85 kcal·mol^−1^
Trypsin—Matched Cyclic Peptide (3avb)	–43.79 kcal·mol^−1^
FBP11 WW Domain (1ywi)	–30.06 kcal·mol^−1^
FE65 WW Domain (2ho2)	–32.02 kcal·mol^−1^
**Overall Performance**	
Putative match found	82 %
Stable binding during MD	86 %
Average F-RMSD of best matches	0.38 Å
Average L-RMSD of best matches	2.77 Å
Average MM/GBSA of best matches	−43.09 kcal·mol^−1^

Next we applied our cPEPmatch approach systematically to 154 protein-protein complexes with known structures to identify putative matches that could potentially bind at the PP interface and interfere with PP interaction. According to our threshold criteria (see Methods) we were able to identify at least one putative cyclic peptide match for 82% of all the cases studied (Table 1). The putative cyclic peptides matched with the best RMSD and sterical fit to each PPI were superimposed, interface residues were substituted and the interface structure was refined by short MD simulations in explicit solvent. We found that during the 5 ns of MD, 86% of the matched complexes were stable (no dissociation and L-RMSD < 5 Å). Hence, the overall yield of stable predicted complexes of cyclic peptides with protein binding partners was ~71% (0.82*0.86). These identified complex structures had an average F-RMSD, L-RMSD and MMGBSA interaction energy of 0.38 Å, 2.77 Å, and –43.09 kcal mol^−1^, respectively.

**Figure 3:**
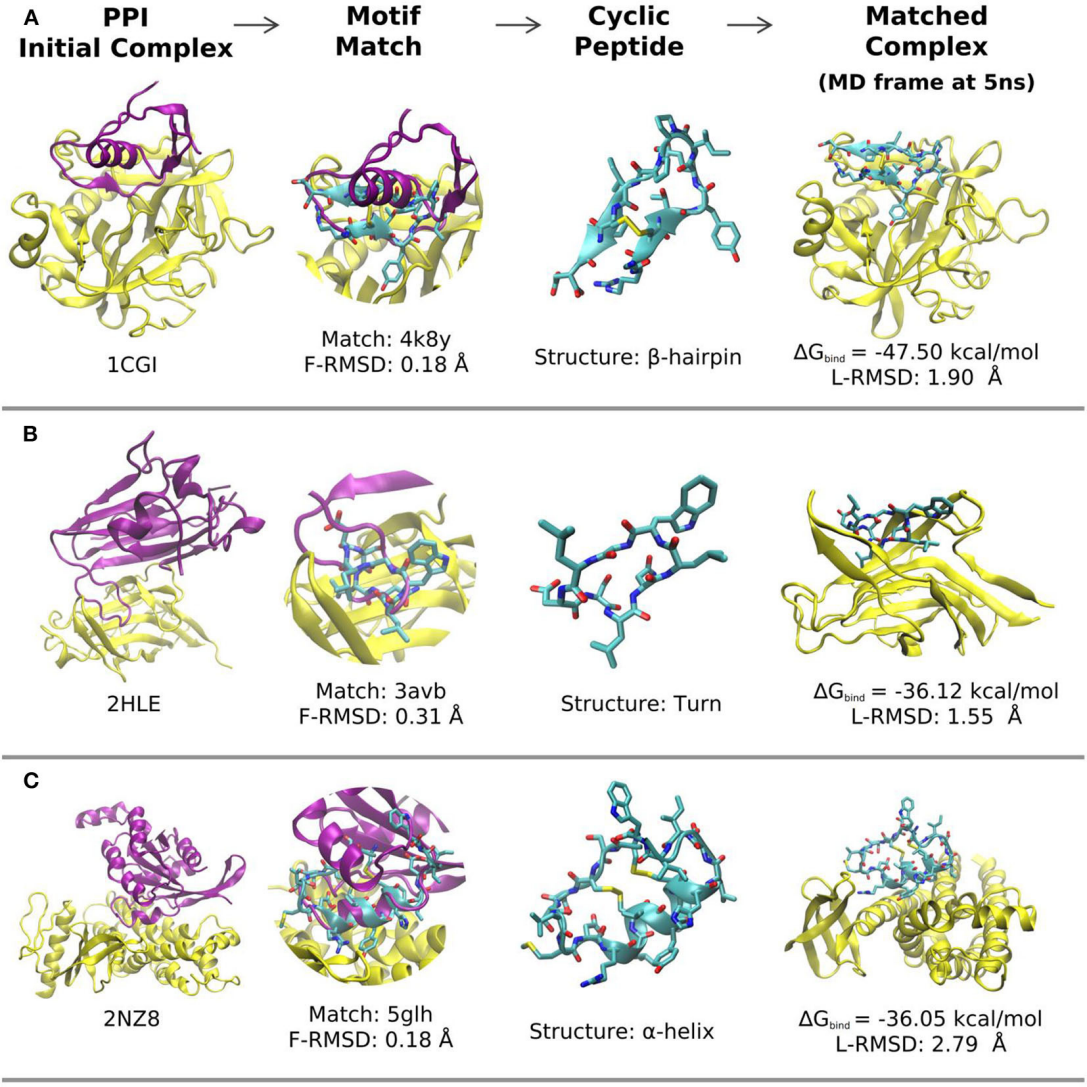
Representative matches and modeled structures of protein-cyclic-peptide complexes. **(A)** Example of a cyclic peptide with an β-hairpin motif at the interface (pdb-entries of the complex and the cyclic peptide template are indicated; the calculated MMGBSA interaction energy and final deviation from the start structure are also included). **(B)** Same as in **(A)** but for a cyclic peptide with a turn motif as binder. **(C)** Same as in **(A,B)** for a cyclic α-helix binding motif. In each case the target protein-protein complex is shown as cartoon (yellow: receptor; pink: ligand protein). The superimposed matching cyclic peptide is indicated in the second column and the cyclic peptide (with adapted interface sequence) is shown in the third column. The last column represents the final structures of the receptor protein (yellow) in complex with the cyclic peptide after 5 ns MD simulation in explicit solvent.

Examples of predicted complexes between cyclic peptides that mimic a β-hairpin, a turn and an α-helical interface segment and partner proteins pdb1cgI, pdb2hle, and pdb2nz8, respectively, are illustrated in Figures 3A–C. A more detailed list of the top 30 matches in terms of calculated MMGBSA interaction energy is found in Table 2. The L-RMSD of all these complexes is in between 1 and 5 Å. The calculated MM/GBSA interaction energies vary significantly from system to system (from ~ –80 to –30 kcal mol^−1^) which might be due to different sizes but may also be due to the targeted interface region in each complex. However, the MMGBSA interaction energies are in most cases more favorable compared to small linear peptides that binding with micromolar binding constants to target domains (Table 1).

**Table 2:** Results for the 30 top matches.

**PPI**	**Cyclic peptide**	**F-RMSD**	**MM/GBSA**		**L-RMSD**
		**(Å)**	**ΔGbind (kcal/mol)**		**(Å)**
1E96	2lwu	1.56	–66.46	±0.52	3.53
1ACB	4k8y	0.11	–50.78	±0.33	1.17
1AHW	1qx9	2.38	–29.30	±0.54	3.42
1AZS	5glh	0.17	–46.10	±0.33	3.98
1BKD	5glh	0.07	–82.38	±0.46	2.43
1CGI	4k8y	0.18	–47.50	±0.34	1.90
1D6R	4k8y	0.09	–53.57	±0.60	2.07
1E6E	5glh	0.12	–42.75	±0.28	3.95
1EAW	4k8y	0.43	–49.78	±0.35	2.23
1FAK	6pio	1.44	–36.27	±0.59	2.73
1FSK	3p8f	0.36	–40.38	±0.29	2.36
1K74	5glh	0.34	–39.63	±0.33	4.77
1LFD	1ebp	0.43	–30.43	±0.33	4.00
1NW9	5glh	0.25	–31.99	±0.43	4.51
1PXV	4k8y	0.18	–40.25	±0.30	3.99
1R0R	4k8y	0.10	–40.74	±0.38	1.89
1VFB	5glh	0.18	–34.36	±0.24	3.23
2B4J	1npo	0.13	–29.26	±0.25	4.16
2C0L	5glh	0.05	–54.09	±0.51	1.77
2CFH	5glh	0.18	–50.49	±0.41	2.53
2FD6	2lwt	0.75	–37.97	±0.47	1.83
2HLE	3avb	0.16	–36.12	±0.25	1.55
2NZ8	5glh	0.18	–36.05	±0.33	2.79
2SNI	3p8f	0.44	–57.30	±0.26	1.11
2W9E	5glh	0.11	–40.54	±0.34	4.85
3AAA	5glh	0.09	–43.83	±0.39	1.35
3BP8	3p8f	0.47	–36.02	±0.23	3.41
3EOA	4k8y	0.23	–41.55	±0.28	1.27
3L5W	5glh	0.09	–30.03	±0.27	1.02
3V6Z	5glh	0,17	–36.90	±0.52	3.38

**Figure 4:**
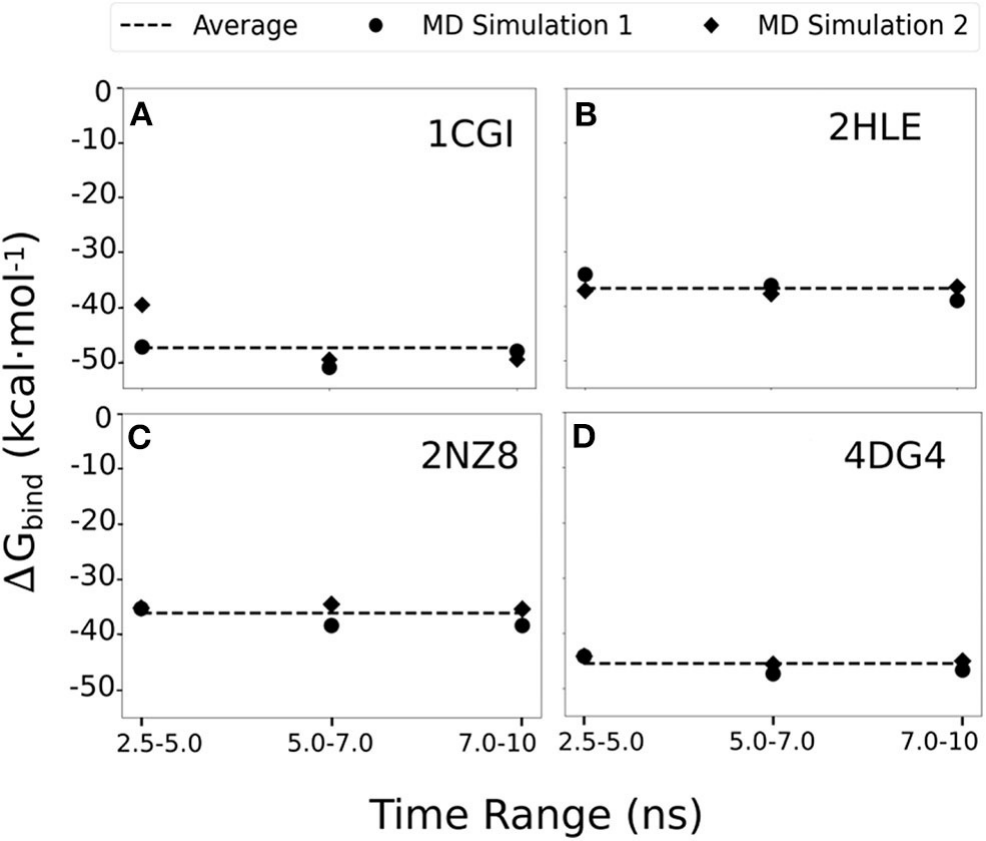
MMGBSA binding free energy calculations estimated using 250 snapshots retrieved from 2.5 to 5.0, 5.0 to 7.0, and 7.5 to 10 ns of two independent MD simulation productions for each of the studied systems **(A)** 1cgi, **(B)** 2hle, **(C)** 2nz8, and **(D)** 4dg3. The average binding free energy for each case is shown as a dashed line along the graphs.

To assess the consistency of our simulations and MMGBSA evaluation, we expanded our original MD productions by 5 ns and also performed a second independent set of MD simulations (of 10 ns) for each of the highlighted systems in our study, 1cgi, 2hle, 2nz8, and 4dg4 (that represent typical β-hairpin, a turn and an α-helical interfaces). For each case and each simulation MMGBSA binding free energy calculations were calculated using 250 snapshots retrieved from the three time intervals corresponding to 2.5–5.0, 5.0–7.0, and 7.5–10 ns of every simulation (Figures 4A–D). Overall, the calculated MMGBSA binding energies are close to the average for each case with a standard error of < 0.8 kcal mol^−1^ even when considering both independent simulations for each case supporting the robustness of the evaluation protocol.

**Figure 5:**
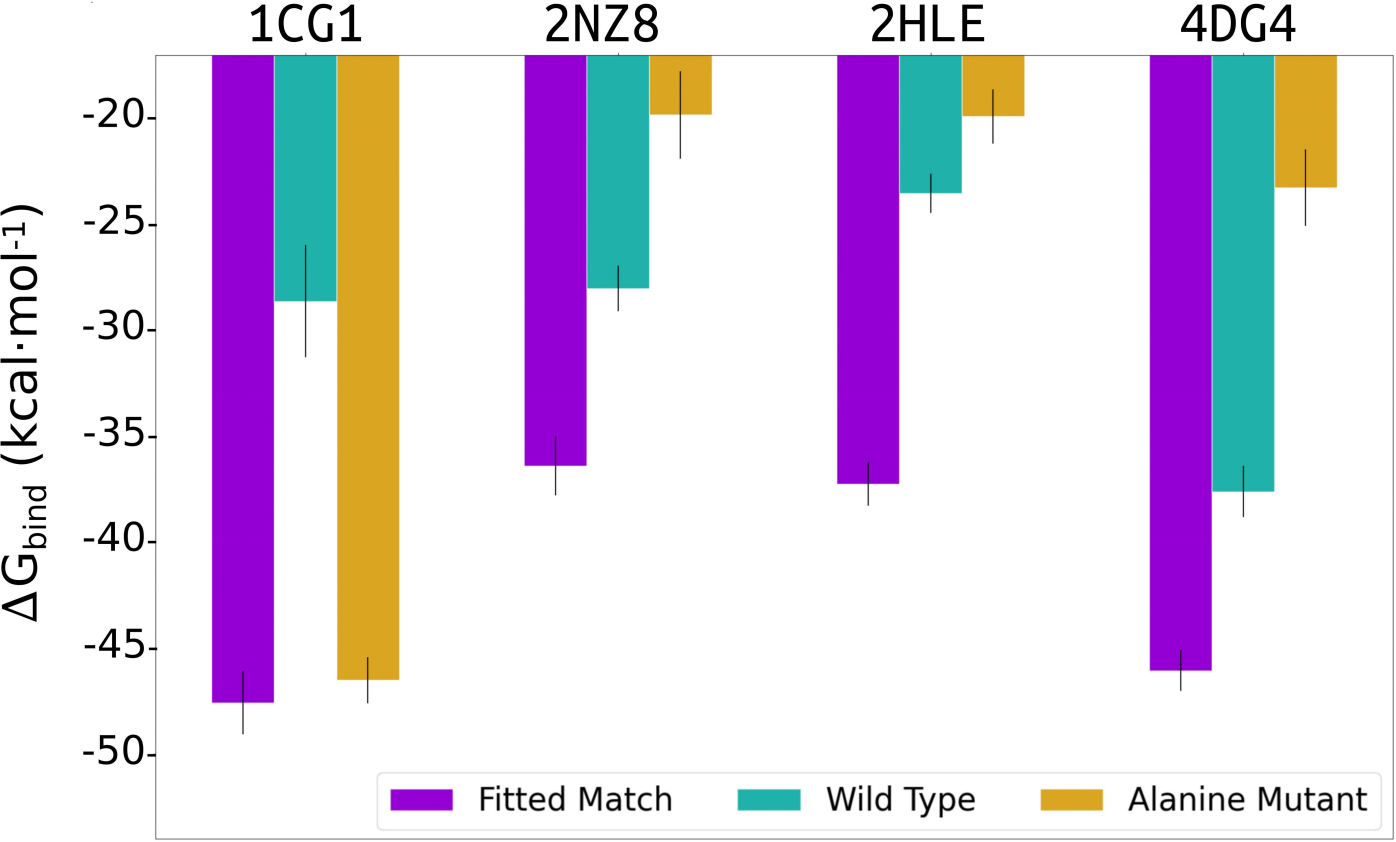
Average of 3 MMGBSA binding energy calculations estimated using 250 snapshots retrieved from three intervals (2.5–5.0, 5.0–7.0, and 7.5–10 ns) of MD simulations for the 1cgi, 2nz8, 2hle, and 4dg4 PPI receptors in complex with the cPEPmatch cyclic peptides and interface residues copied from the native complex (purple bar), or using the original cyclic peptide sequence (wild type) from the data base (cyan bar) or alanine substitutions for all interface residues of the cyclic peptide (orange bar).

In addition, we evaluated the importance of the generated interface side chains on the ligand-fitted cyclic peptides constructed by our cPEPmatch approach that are identical to the side chains of the native complex. Our evaluation procedure was applied to protein bound cyclic peptides containing the original sequence of the corresponding cyclic peptide from the data base or containing just alanine substitutions at the interface (Figure 5). The same set of representative cases (1cgi, 2cfh, 2hle, and 4dg4) were used. The cyclic peptides with alanines at the interface proved to be considerably weaker binders in our MMGBSA evaluation (Figure 5) compared to the cyclic peptides with native interface residues when the secondary structure is an α-helix or a turn (2hle, 2nz8, and 4dg4). However, for the case with the cyclic peptide providing a β-hairpin interface (1cgi) predicted binding was almost as strong as for the native sequence (Figure 5). In this case, however, a significant amount of backbone-backbone interactions that take place at the β-hairpin interfaces was observed which may not be perturbed by the alanine substitutions. Whereas, in three out of the four test cases the alanine substitution reduced the MMGBSA score using the original sequence of the cyclic peptides (from the data base) gave in all cases a strong reduction of the calculated MMGBSA binding energy (Figure 5, green bars, labeled wild type).

## Conclusions

One possibility to create cyclic peptide variants based on known protein-protein interface structures is to identify a linear interface motif with an appropriate distance of terminal side chains or backbone atoms such that cyclization is possible. Ideally, such motifs give stable cyclic peptide binders. However, it can be difficult to predict the backbone structure of a cyclized peptide if one introduces a chemical backbone or side chain crosslink. The crosslink can destabilize a desired geometry such that the cyclic peptide does not resemble the structure of the interface motif. An advantage of our strategy is that we base the construction of a desired cyclic peptide on known stable (high resolution) cyclic template structures. Hence, the approach avoids the uncertainty on how well a select cyclization of a given motif resembles a desired backbone structure. It is applicable if the structure of a protein-protein complex is known and even potentially useful if the receptor is flexible and adopts a different structure in the unbound form (as long as it can adopt the structure in the bound form). Most related to our method is an approach by Duffy et al. (2015) to identify both protein–protein interactions suitable for inhibition by cyclic peptides and the accompanying cyclic peptides that represent promising lead compounds. This approach is, however, based on a pharmacophore matching of the PPI interface region with corresponding side chains on sets of cyclic peptides whereas our method is entirely based on structural matching of the interface backbone onto segments of cyclic peptides with known structure. It is complementary also to other approaches to identify cyclic peptides that target PPI (Schreiber and Crabtree, 1992; Dechantsreiter et al., 1999; Sulyok et al., 2001; Shi et al., 2004; Zhang et al., 2014; Siegert et al., 2016; Shin et al., 2017).

The initial screen of our approach (without explicit solvent refinement) is extremely rapid, hundreds of PPI can be screened within a few seconds for cyclic peptides that match to backbone structures at the PPI interface. Interestingly, with a relatively small set of cyclic peptide templates it is possible to identify cyclic peptide-protein complexes that are stable during explicit solvent MD simulations and indicate very favorable interaction energy scores comparable or better than for known stable peptide-protein complexes. We also found that substitution by alanine in the evaluation could be a useful strategy in order to check the contribution of the side chains copied from the native interface to the cyclic peptide-protein interface to the stability of the complexes. It should be noted that a short MD simulation as used in our approach is useful to relax the complex structure but not long enough to exhaustively sample the complex conformations. The putative cyclic peptide modulators could be further improved by individual post-modifications and by studying specific cases experimentally. It could also be combined with approaches to identify peptide epitopes specific for mediating protein-protein interactions as well as allosteric sites our unstable loop segments in protein interaction regions (Paladino et al., 2020; Serapian and Colombo, 2020). Disruption of interactions that involve such regions often interferes with specific functions of the corresponding protein-protein complex. Our cPEPmatch method, although at an early stage, has demonstrated the ability to be an easy automatic step toward identifying putative cyclic peptide templates for PPI modulators that could form the basis for subsequent experimental testing or more extensive analysis of the interface including the hydration properties. In the future, a larger and even more diverse set cyclic peptides will be constructed and some of the putative cyclic peptides will be tested experimentally.

## Author Contributions

MZ designed and supervised research. BS performed research and analyzed data. All authors contributed to writing of the manuscript.

## Conflict of Interest

The authors declare that the research was conducted in the absence of any commercial or financial relationships that could be construed as a potential conflict of interest.
