# Supplementary material for: Rapid in silico Design of Potential Cyclic Peptide Binders Targeting Protein-Protein Interfaces
Source: Front Chem. 2020 Oct 8;8:573259. doi: 10.3389/fchem.2020.573259 (PMC7578414; doi:10.3389/fchem.2020.573259)
Supplement: Supplementary file 1 [file Data_Sheet_1.PDF]

Supplementary Material:

Rapid in silico design of potential cyclic peptide binders targeting protein-protein interfaces

Brianda L Santini, Martin Zacharias*

Physics Department T38, Technical University of Munich, James-Franck-Straße 1, 85748 Garching, Germany

Table S1. Cyclic peptide library.

PDB	Type of Cyclization	Residues
1ebp	Single Disulfide Bond	16
1foz	Head to Side Chain	7
1jbl	Head to Tail + Single Disulfide Bond	14
1npo	Single Disulfide Bond	9
1qx9	Single Disulfide Bond	16
1sld	Single Disulfide Bond	6
2ajw	Head to Tail + Double Disulfide Bond	22
2ak0	Head to Tail + Double Disulfide Bond	23
2jrw	Single Disulfide Bond	23
2lws	Head to Tail + Single Disulfide Bond	12
2lwt	Head to Tail + Single Disulfide Bond	18
2lwu	Head to Tail + Single Disulfide Bond	15
2lwv	Head to Tail + Single Disulfide Bond	17
2m2g	Head to Tail + Double Disulfide Bond	18
2n07	Head to Tail + Single Disulfide Bond	22
2ox2	Head to Tail	6
3avb	Head to Tail	8
3p8f	Head to Tail + Single Disulfide Bond	14
3wne	Head to Tail	6
3wnf	Double Disulfide Bond	6
4k8y	Single Disulfide Bond	13
5glh	Double Disulfide Bond	21
5lff	Single Disulfide Bond	7
5vav	Head to Tail + Single Disulfide Bond	14
6awk	Head to Tail	8
6awm	Head to Tail	7
6axi	Head to Tail	8
6pin	Head to Tail + Double Disulfide Bond	18
6pio	Head to Tail + Double Disulfide Bond	18
6pip	Head to Tail + Double Disulfide Bond	18

Collection of the cyclic peptides used as templates for the design strategy. The selected structures are high resolution crystal structures and vary in sizes from 6 to 23 amino acids, represent different types of cyclizations: 6 by head to tail, 1 by head to side-chain, 7 by a single disulfide bond, 2 by double disulfide bonds and 14 include combined cyclizations.

Table S2. Protein-protein interaction complexes chosen for testing together with their resulting best matched structure.

PPI	Match	F-RMSD (Å)	L-RMSD (Å)	PPI	Match	F-RMSD (Å)	L-RMSD (Å)	PPI	Match	F-RMSD (Å)	L-RMSD (Å)
**1A2K**	1ebp	0,21	5,17	**1KKL**	1ebp	0,21	14,29	**2G77**	4k8y	0,47	1,15
**1ACB**	4k8y	0,11	1,17	**1KTZ**	N/M	/	/	**2H7V**	N/M	/	/
**1AHW**	1qx9	2,38	3,42	**1KXQ**	1ebp	0,14	6,47	**2HLE**	3avb	0,16	1,55
**1AK4**	N/M	/	/	**1LFD**	1ebp	0,43	4,00	**2HMI**	1ebp	0,12	4,59
**1AVX**	4k8y	0,19	1,64	**1M10**	2lwt	2,27	14,66	**2HQS**	5glh	0,26	5,13
**1AY7**	3wne	0,09	3,08	**1M27**	N/M	/	/	**2HRK**	5glh	0,08	4,00
**1AZS**	5glh	0,17	3,98	**1MAH**	4k8y	0,20	5,68	**2I25**	1ebp	0,48	9,53
**1B6C**	5glh	0,07	6,71	**1ML0**	3p8f	0,22	3,34	**2IDO**	5glh	0,07	7,70
**1BJ1**	1qx9	2,02	3,27	**1MLC**	1ebp	0,14	8,39	**2J0T**	3p8f	0,31	4,26
**1BKD**	5glh	0,07	2,43	**1MQ8**	5glh	0,22	11,45	**2JEL**	1ebp	0,12	1,83
**1BUH**	N/M	/	/	**1NSN**	2ajw	1,84	8,69	**2MTA**	5glh	0,20	7,56
**1BVK**	N/M	/	/	**1NW9**	5glh	0,25	4,51	**2NZ8**	5glh	0,18	2,79
**1BVN**	4k8y	0,35	3,45	**1OFU**	4k8y	0,10	4,70	**2O3B**	2lwv	3,27	2,50
**1CGI**	4k8y	0,18	1,90	**1OPH**	3p8f	0,33	51,07	**2O8V**	5glh	0,15	4,00
**1D6R**	4k8y	0,09	2,07	**1OYV**	3avb	0,11	4,46	**2OOB**	5glh	0,05	10,53
**1E6E**	5glh	0,12	3,95	**1PPE**	3p8f	0,18	4,53	**2OOR**	5glh	0,13	4,04
**1E6J**	N/M	/	/	**1PVH**	5glh	0,09	6,58	**2OT3**	4k8y	0,08	2,12
**1E96**	2lwu	1,56	3,53	**1PXV**	4k8y	0,18	3,99	**2OUL**	3avb	0,15	4,76
**1EAW**	4k8y	0,43	2,23	**1QA9**	N/M	/	/	**2PCC**	N/M	/	/
**1EFN**	5glh	0,12	4,83	**1QFW**	1ebp	0,19	5,27	**2SIC**	N/M	/	/
**1EWY**	4k8y	0,33	6,62	**1R0R**	4k8y	0,10	1,89	**2SNI**	3p8f	0,44	1,11
**1F6M**	N/M	/	/	**1R6Q**	5glh	0,30	5,58	**2UUY**	4k8y	0,18	3,75
**1FAK**	6pio	1,44	2,73	**1R8S**	5glh	0,15	4,23	**2VDB**	5glh	0,08	3,10
**1FC2**	5glh	0,06	5,12	**1RV6**	1qx9	2,25	9,69	**2VIS**	1ebp	0,13	15,42
**1FFW**	5glh	0,12	5,15	**1S1Q**	3p8f	0,27	8,35	**2VXT**	3avb	0,21	2,84
**1FLE**	N/M	/	/	**1SBB**	1ebp	0,32	5,01	**2W9E**	5glh	0,11	4,85
**1FQ1**	5glh	0,05	1,22	**1SYX**	5glh	0,09	7,59	**2X9A**	1ebp	0,12	5,68
**1FQJ**	2ajw	1,67	28,05	**1T6B**	N/M	/	/	**2Z0E**	1ebp	0,19	3,84
**1FSK**	3p8f	0,36	2,36	**1TMQ**	5glh	0,09	5,59	**3A4S**	6pio	2,31	18,29
**1GCQ**	3p8f	0,11	4,54	**1UDI**	3p8f	0,13	2,26	**3AAA**	5glh	0,09	1,35
**1GHQ**	2lwt	2,14	4,30	**1US7**	5glh	0,08	10,06	**3BIW**	5glh	0,26	8,96
**1GL1**	3p8f	0,29	8,09	**1VFB**	5glh	0,18	3,23	**3BP8**	3p8f	0,47	3,41
**1GLA**	N/M	/	/	**1WEJ**	N/M	/	/	**3BX7**	N/M	/	/
**1GP2**	N/M	/	/	**1WQ1**	N/M	/	/	**3CPH**	4k8y	0,10	2,34
**1GRN**	5glh	0,07	5,31	**1XQS**	5glh	0,09	4,53	**3D5S**	5glh	0,10	4,76
**1GXD**	5glh	0,31	2,89	**1XU1**	N/M	/	/	**3DAW**	5glh	0,11	4,56
**1H1V**	5glh	0,09	4,77	**1YVB**	1ebp	0,25	3,78	**3EOA**	4k8y	0,24	5,28
**1HCF**	N/M	/	/	**1Z0K**	5glh	0,12	5,61	**3K75**	1ebp	0,37	7,56
**1HE1**	5glh	0,06	19,75	**1Z5Y**	1ebp	0,29	3,04	**3L5W**	5glh	0,09	2,29
**1HE8**	1ebp	0,23	8,23	**1ZHI**	1ebp	0,11	10,41	**3LVK**	N/M	/	/
**1HIA**	1ebp	0,31	1,04	**1ZM4**	4k8y	0,29	3,64	**3P57**	5glh	0,13	5,14
**1IB1**	5glh	0,14	4,67	**2A5T**	5glh	0,10	8,50	**3PC8**	4k8y	0,26	8,08
**1IJK**	N/M	/	/	**2A9K**	5glh	0,05	31,02	**3SGQ**	4k8y	0,39	4,62
**1IQD**	N/M	/	/	**2ABZ**	5glh	0,08	4,52	**3V6Z**	5glh	0,17	3,38
**1J2J**	5glh	0,05	4,35	**2AJF**	5glh	0,10	5,15	**4DN4**	5glh	0,11	7,07
**1JIW**	N/M	/	/	**2AYO**	3p8f	0,14	8,15	**4G6M**	3p8f	0,24	3,26
**1JK9**	N/M	/	/	**2B4J**	1npo	0,13	4,16	**4GXU**	N/M	/	/
**1JMO**	6pin	3,05	6,70	**2BTF**	5glh	0,10	6,51	**4IZ7**	5glh	0,06	5,66
**1JZD**	3p8f	0,32	5,92	**2C0L**	5glh	0,05	1,77	**7CEI**	5glh	0,17	8,35
**1K4C**	5glh	0,15	25,43	**2CFH**	5glh	0,18	2,53	**9QFW**	N/M	/	/
**1K74**	5glh	0,34	4,77	**2FD6**	2lwt	0,75	1,83				
**1KAC**	5glh	0,22	9,25	**2FJU**	N/M	/	/				

N/M: indicates no matching cyclic peptide motif found.
